# Elevated autocrine EDIL3 protects hepatocellular carcinoma from anoikis through RGD-mediated integrin activation

**DOI:** 10.1186/1476-4598-13-226

**Published:** 2014-10-01

**Authors:** Ming-Xuan Feng, Ming-Ze Ma, Ying Fu, Jun Li, Tao Wang, Feng Xue, Jian-Jun Zhang, Wen-Xin Qin, Jian-Ren Gu, Zhi-Gang Zhang, Qiang Xia

**Affiliations:** Department of Liver Surgery, Renji Hospital, School of Medicine, Shanghai Jiao Tong University, Shanghai, 160 Pujian Road, Shanghai, 200127 China; State Key Laboratory of Oncogenes and Related Genes, Shanghai Cancer Institute, Renji Hospital, Shanghai Jiao Tong University School of Medicine, 800 Dongchuan Road, Shanghai, 200240 China

**Keywords:** Epidermal growth factor-like repeats and discoidin I-like domains 3, Hepatocellular carcinoma, Anoikis resistance, Integrin activation

## Abstract

**Background:**

A remolded microenvironment in hepatocellular carcinoma (HCC) caused by abnormally expressed matricellular proteins could promote HCC progression. The cell-matrix interactions mediated by integrins play an important role in tumor microenvironment. Epidermal Growth Factor-like repeats and Discoidin I-Like Domains 3 (EDIL3), an extracellular matrix (ECM) protein with angiogenic and anti-inflammatory effects, is abnormally highly expressed in HCC. Here we aim to analyze its expression in liver and HCC tissues, investigate the underlined mechanisms accounted for HCC progression.

**Methods:**

EDIL3 expression level is examined in normal liver, cirrhotic liver and HCC at both mRNA and protein level. The association between EDIL3 and clinical outcomes is analyzed. The pattern of EDIL3 expression and location is examined using Immunofluorescence and ELISA. Overexpression or knock-down of EDIL3 in a panel of cell lines are subjected to assays related to proliferation, invasion, and anoikis to investigate the mechanisms of this matrix protein in HCC progression. Recombinant EDIL3 treatment is applied to confirm the results.

**Results:**

Compared with normal liver and cirrhotic liver, EDIL3 is elevated in HCC. High level of EDIL3 protein is much more commonly in patients with larger tumor or portal vein tumor thrombus (PVTT) formation, associated with poor prognosis. EDIL3 is abundantly expressed in HCC cells and secreted by cancer cells. *In vitro* and *in vivo* studies indicate that EDIL3, probably in an autocrine manner, inhibits anoikis and promotes anchorage-independent growth of HCC cells. Further mechanistic studies suggest integrin ligation by EDIL3 and thus that the sustained activation of the FAK-Src-AKT signal is responsible for the anoikis resistance and anchorage independence. Both the administration of cilengitide, a RGD-containing integrin antagonist, and silencing of integrin αV, an important RGD-binding integrin, results in the blockade of anoikis-resistance induced by EDIL3.

**Conclusion:**

Our study suggests that high levels of autocrine EDIL3 may contribute to a receptive microenvironment for the survival of detached HCC cells and may involve in cancer cell spreading. We also highlight the importance of interaction between EDIL3 and integrin αV and suggest disrupting the ligation of EDIL3 to integrins via RGD-blocking in selected patients may bear potential therapeutic value.

**Electronic supplementary material:**

The online version of this article (doi:10.1186/1476-4598-13-226) contains supplementary material, which is available to authorized users.

## Introduction

Hepatocellular carcinoma (HCC) is a lethal disease with high mortality due to the high rate of postoperative recurrence and metastasis [1]. Both genetic and epigenetic alterations [2] within the HCC and a remolded microenvironment [3, 4] surrounding HCC affect the biological behaviors of HCC and thus result in different outcomes of HCC patients. Extracellular matrix (ECM) proteins are the main non-cellular components of the tumor microenvironment; many of them can interact with the tumor cell through direct binding to specific receptors or by modulating the activation of other cytokines [5, 6]. Some reports have suggested that microenvironmental changes caused by abnormally expressed matricellular proteins may modulate HCC progression by affecting cell growth, migration, invasion, anoikis and metastasis; thus, targeting ECM proteins may have therapeutic value [3, 7–10].

Epidermal Growth Factor-like repeats and Discoidin I-Like Domains 3 (EDIL3), also known as DEL-1, is a secreted ECM protein that was firstly characterized in vascular morphogenesis [11]. Structurally, EDIL3 contains 3 EGF-like repeats and 2 discoidin-like repeats, with an RGD motif located in the second EGF-like repeat [11]. Three-dimensional crystal structures reveal a unique RGD finger, which is believed to be advantageous for its ligation with integrins over other RGD-containing ECM proteins [12]. EDIL3 has been intensively studied in vascularization, exhibiting strong angiogenic or vascularizing function through integrin αvβ3 modulation [13, 14]. Moreover, this protein plays a role in modulating immunocyte adhesion through interactions with leukocyte-specific integrins [15]. The role of EDIL3 in cancer is also revealed in several malignancies, albeit in observational level. For example in pancreatic carcinoma, EDIL3 is one of the SHH-dependent stromal factors that predict poor prognosis [16]; another study focusing on carcinogenesis of ulcerative colitis-associated colorectal cancer suggests EDIL3 may add some power in this process [17]; interestingly, EDIL3 could also be detected in exosomes of bladder cancer cells and facilitate cancer progression trough EGFR signal [18]. In the field of HCC, high-throughput genomic data suggests elevated EDIL3 in HCC compared with adjacent liver [19], and a clinical analysis reveals that EDIL3 might affect the prognosis of HCC [20]. However, no study has addressed how EDIL3 is involved in HCC development and progression.

Anoikis is a special apoptotic process resulting from the loss of or inappropriate cell adhesion. Upon detaching from the primary lesion, the lack of cell-ECM adhesion fails to maintain the pro-survival signals within cancer cells, leading to decreased anti-apoptotic signals, thus activating anoikis [21]. Gaining anoikis resistance is a prerequisite for intra-hepatic spreading and extra-hepatic metastasis of HCC. In addition to adaptive alterations, such as switch in integrin expression patterns or the hyperactivation of receptor tyrosine kinases, the abnormal microenvironment also helps the cancer evading anoikis [22]. Integrins are the key mediators of the cell-ECM interaction, sending pro-survival signals in the presence of ECM ligands, e.g., collagens and laminins, and inducing apoptosis in their unligated status [23]. EDIL3 is an important ligand for αV-coupled integrins via RGD recognition and has been shown to be able to bind these groups of integrins [24] and inhibit anoikis in the endothelium [25].

In the present study, we focus on the role of EDIL3 in HCC and demonstrate that EDIL3 is highly expressed in HCC patients. Moreover, the accumulation of tumor-derived EDIL3 in the microenvironment promotes anoikis resistance and anchorage independent growth advantage through the activation of integrin signal pathways.

## Results

### The EDIL3 level is significantly elevated in HCC tissue and is associated with adverse biological behaviors

**Figure 1:**
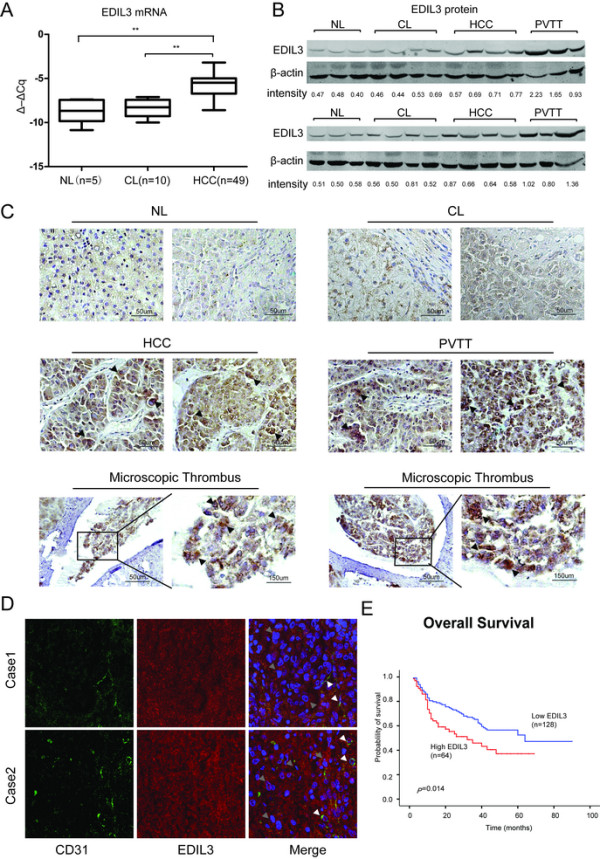
**EDIL3 is significantly up-regulated in HCC and PVTT compared with normal liver (NL) and cirrhotic livers (CL) and is closely related to the prognosis of HCC. A**, The transcriptional level of EDIL3 is measured via qRT-PCR in 5 NLs, 10 CLs and 49 HCCs. The relative mRNA level is normalized to β-actin and is presented as Δ–ΔCq. The mRNA of EDIL3 in HCC is significantly higher than both NL and CL; **B**, The protein level of EDIL3 in NL, CL, HCC and PVTT was examined by western blot with β-actin as a loading control. The EDIL3/β-actin densitometry is performed and shown as density value below. EDIL3 is mildly expressed In NL and CL while significantly elevated in HCC and PVTT; **C**, Representative pictures demonstrating EDIL3 staining in different liver samples including NL, CL, HCC, PVTT and microscopic thrombi by IHC. Scale bars, 50 μm or 150 μm; Arrow heads indicate that high EDIL3 expression is largely localized to cancer cells. **D**, Confocal microscopic observation of immunofluorescence staining of CD31 (green), an endothelium marker, and EDIL3 (red) in HCC samples show EDIL3 is not only localized with endothelium, but also widely and diffusively located in cancer cells clusters. White arrows indicates the endothelium; Grey arrows indicates the cancer cell cluster. **E**, Kaplan-Meier analysis of overall survival between EDIL3-negative or moderately positive patients and highly positive patients shows a significant survival advantage in EDIL3 low express group. **: P < 0.01.

To observe the expression change in HCC, we first tested the EDIL3 level in 5 normal livers (NL), 10 cirrhotic livers (CL), and 49 HCCs by qRT-PCR and western blot. The result revealed that EDIL3 was mildly expressed in NLs and CLs but was significantly elevated in HCC both at the mRNA and protein level (Figure 1 A, B, Additional file 1: Figure S1A). IHC stain of another array of samples including 3 NLs, 10HCCs and 6 PVTTs confirmed the high expression of EDIL3 in HCC compared with NL and CL (Figure 1C). Notably, co-stain of EDIL3 and endothelium marker CD31 in HCC samples showed EDIL3 is not only stained with endothelium marker, but also widely and diffusively located in cancer cells clusters (Figure 1C, D), suggesting that cancer cells becomes an important sources of EDIL3 besides endothelium.

We then explored the clinical value of EDIL3 in HCC by analyzing the protein level in another independent 202 HCC samples gathered on tissue microarrays (TMA). We observed approximately 72% (57/202) of patients were EDIL3-positive, and high levels of EDIL3 positivity was significantly associated with adverse clinicopathological parameters of HCC, including tumor size, PVTT formation, and TNM stage (Table 1). Moreover, Kaplan-Meier survival analysis in the 192 patients with follow-up demonstrated a higher postoperative recurrence risk and poor survival in patients with high EDIL3 expression. Because high EDIL3 expression was much more common in patients with PVTT, we examined EDIL3 protein levels in 6 PVTT samples. The result revealed that EDIL3 stain was very strong in all of the PVTT samples (6/6) as well as microscopic tumor thrombus (Figure 1C), and this was validated by western blot (Figure 1B). Taken together, these results reveal the high level of EDIL3 in HCC and suggest its association with adverse clinicopathological parameters and poor prognosis.

**Table 1:** **Correlation between EDIL3 and key clinicopathological parameters**

Variables	EDIL3 (n = 202)			
	**High**	**Positive**	**Negative**	**P value**
Age (years)				
≤50	30	43	30	0.829
>50	36	36	27	
Gender				
Female	7	12	9	0.644
Male	59	67	48	
Tumor size				
≤5 cm	29	31	41	**0.003**
>5 cm	37	48	16	
Thrombus				
No	42	65	51	**0.002**
Yes	24	14	6	
Multiplicity				
Single	52	65	51	0.277
Multiple	14	14	6	
TNM stage				
I	31	40	45	**0.010**
II	7	10	6	
III	27	24	6	
IV	1	1	0	

The bold numbers represent the P-values with significant differences.

### EDIL3 is abundantly expressed in HCC cell lines and exhibits unique expression pattern

**Figure 2:**
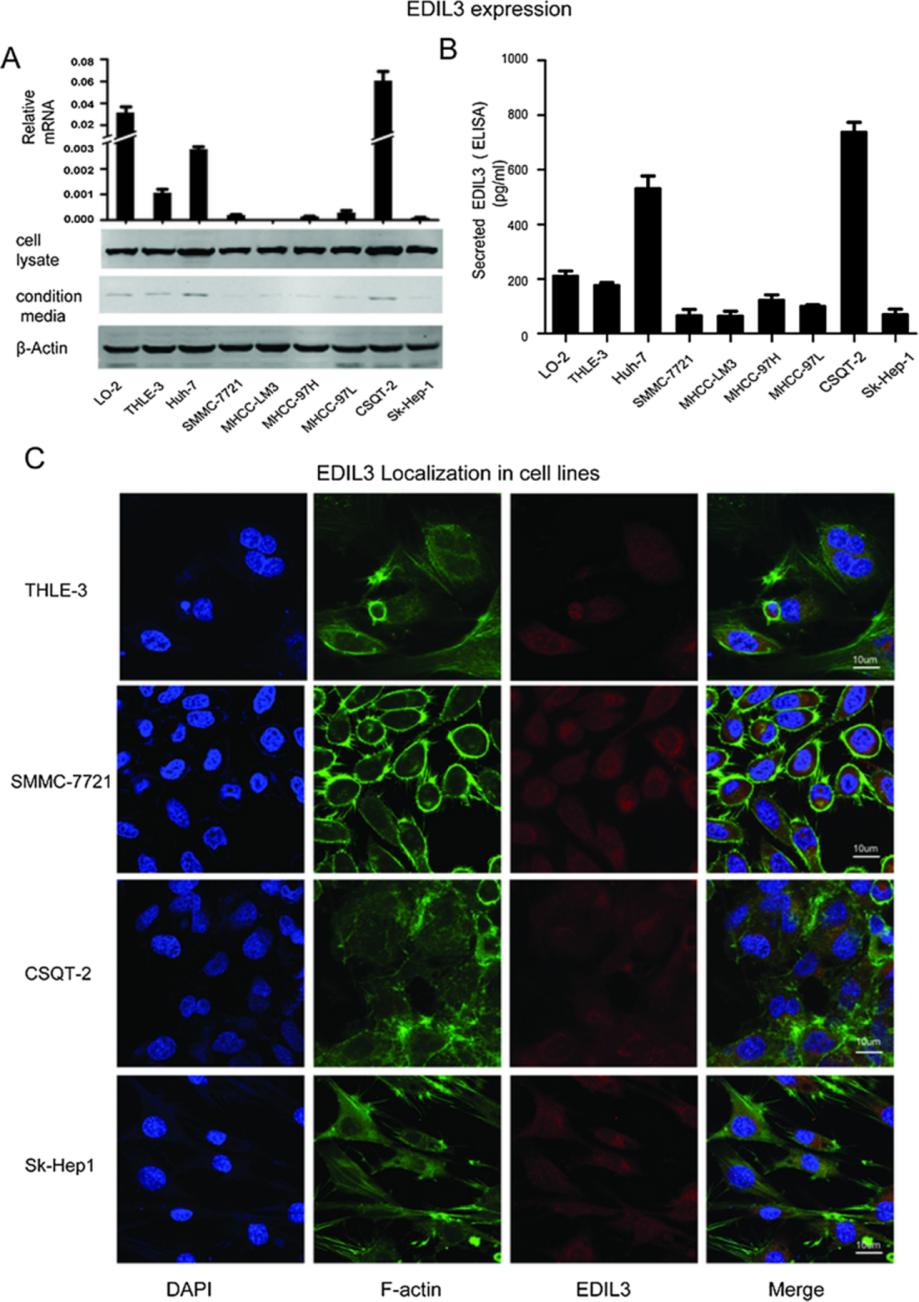
**EDIL3 exhibits a unique expression pattern in cell lines. A**, detailed analysis of EDIL3 expression in mRNA level, cell lysates and CMs of 7 HCC and 2 non-HCC cell lines demonstrated an approximate correlation between mRNA and secreted EDIL3 in CMs, whereas EDIL3 in cell lysates exhibited almost same intensity. Notably, Huh-7 and CSQT-2 exhibited a much higher level of secreted EDIL3. **B**, ELISA assay testing the EDIL3 in CMs of 9 cell lines validates the secreted EDIL3 level is approximately correlated with mRNA level. **C**, Immunofluorescence staining of EDIL3, F-actin and DAPI in confocal microscope shows EDIL3 is localized within cells and at almost the same intensity in all the 9 cell lines under test, despite their varied mRNA level.

We examined EDIL3 expression in 7 HCC cell lines and 2 non-HCC cell lines. The mRNA level of EDIL3 varied among all of the cell lines, with the HCC cell lines Huh-7 and CSQT-2 exhibiting much higher mRNA levels of EDIL3 (Figure 2A), while THLE-3 and Lo-2, which derived from normal hepatocyte, also showed mild transcription. Surprisingly, inconsistent with mRNA, EDIL3 protein was detected at almost the same level in the cell lysates of all the cell lines (Figure 2A) and the Immunofluorescence confirmed the existence of EDIL3 in cytoplasm of all 9 cell lines (Figure 2C and Additional file 1: Figure S1). Indeed, we also observed this inconsistency in other cancer cell lines (Additional file 2: Figure S2). Because EDIL3 is an ECM protein, we examined the EDIL3 that was secreted in conditioned medium (CM). All cell lines were subjected to standardized condition (see methods for details), and the secreted proteins in the CM was analyzed by Western Blot and ELISA. Not surprisingly, the Huh-7 and CSQT-2 secreted the most EDIL3 into the CM, whereas there was only detectable EDIL3 in the CM of other cell lines (Figure 2A, B). These observations in cell lines reveal a unique autocrine expression pattern of EDIL3, and the high level of EDIL3 in CSQT-2, a PVTT derived cell line, is consistent with the observation in patient’s samples above.

### Autocrine EDIL3 promotes anoikis resistance and anchorage-independent growth in HCC cells *in vitro*

**Figure 3:**
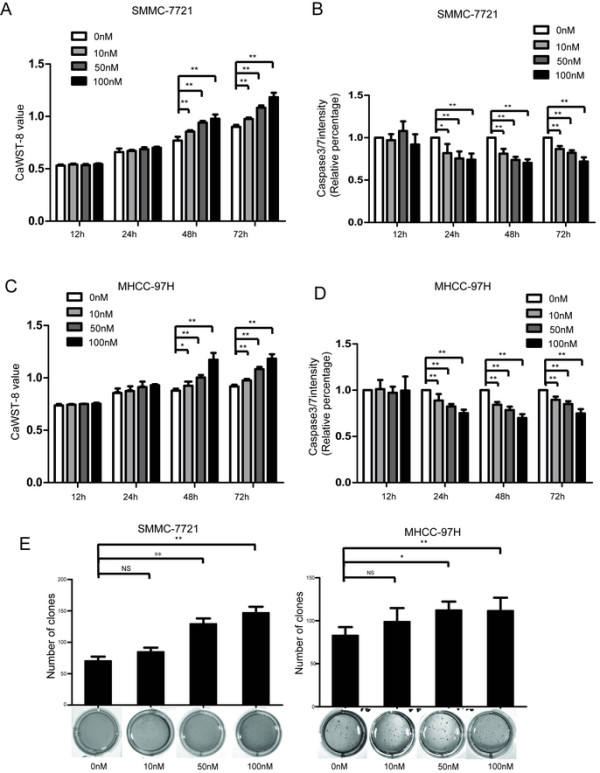
**Treatment of EDIL3 contributes to anoikis resistance and anchorage-independent growth advantage in HCC. A**, Different concentration of recombinant EDIL3 is used to treat SMMC-7721 suspended in poly-hema coated dishes. It sustains the viability of SMMC-7721 as demonstrated by caspase3WSt-8 assay in a time and dose dependent manner compared with control protein. **B**, Recombinant EDIL3 ameliorate the anoikis compared with control protein in SMMC-7721 as demonstrated by caspase3/7 intensity assay in a time and dose dependent manner. **C-D**, MHCC-97H is subjected to the assays same as SMMC-7721 and obtain consistent result. **E**, Administration of recombinant EDIL3 increases the anchorage-independent growth of SMMC-7721 and MHCC-97H cells in soft agar compared with control protein in a dose dependent manner. The assays last for 28 days. *: P < 0.05; **: P < 0.01.

Anoikis resistance and invasion are two important features of the HCC cells that are prone to forming PVTT or metastasis. We continued to investigate the effect of EDIL3 on tumor cell invasion and anoikis. A eukaryotic expression system suitable for secreted protein synthesis was used to generate recombinant EDIL3 with high fidelity (Additional file 3: Figure S3). SMMC-7721 and MHCC-97H, two cell lines with low basal levels of autocrine EDIL3, were chosen to undergo functional assays in the presence of EDIL3. EDIL3 did not affect invasion or proliferation (Additional file 2: Figure S2B). However, using WST-8 and Caspase3/7 activity assay, we found EDIL3 significantly lowered the anoikis rate. EDIL3 treatment reduced the Caspase3/7 activity and increased the viability in cancer suspended in poly-hema coated dishes for long to 72 h (Figure 3A-D). In soft agar clone formation, EDIL3 also significantly promoted the anchorage-independent growth in SMMC-7721 and MHCC-97H cells (Figure 3E). These effects above are both dose-dependent, with the 50 nM safely reaching significance.

**Figure 4:**
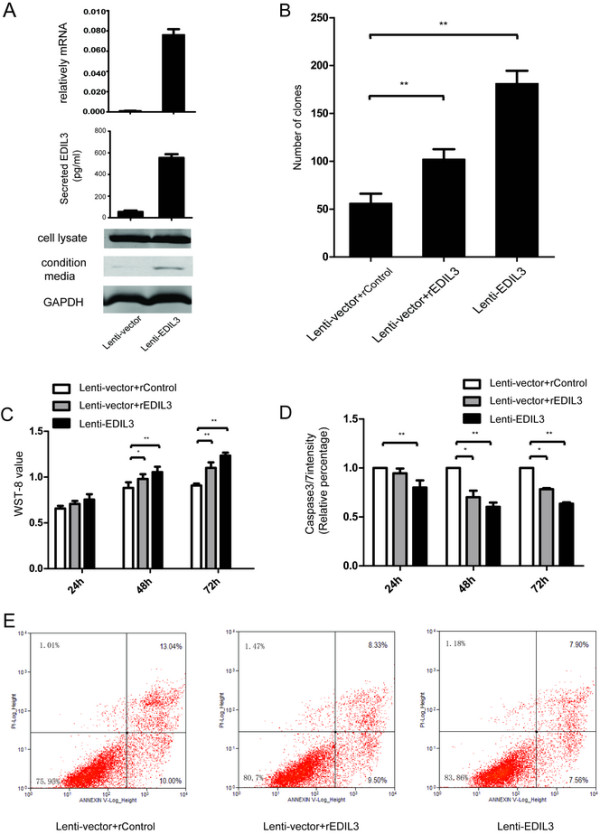
**Overexpression of EDIL3 in SMMC-7721 cells significantly increases the anoikis resistance and anchorage independence. A**, Overexpression of EDIL3 is validated by real-time PCR, western blot and ELISA assay, confirming that the increase in transcription leads to a higher autocrine EDIL3. **B**, EDIL3-overexpressing SMMC-7721 forms more clones in soft agar compared with control cells, which can be partly mimicked by recombinant EDIL3 (50 nM). **C**, After being suspended for up to 72 h, the overexpressing group demonstrates a high WST-8 value compare to the control cells at 48 h and 72 h, and administration of recombinant EDIL3(50 nM) in control cells partly mimics the effect induced by EDIL3 overexpression. **D**, Caspase3/7 intensity assay validate that the difference in cell viability between the two groups is caused by lower apoptosis induction. **E**, Annexin V/PI stain by FACS is performed to validate the apoptosis result. EDIL3 overexpression lowers both the early phase and later phase apoptosis percentage and recombinant protein partly mimics this effect. *: P < 0.05; **: P < 0.01.

Furthermore, we transfected SMMC-7721 and MHCC-LM3 with overexpression vectors. Elevated mRNA of EDIL3 did not increase intracellular EDIL3 levels in western blot; however, we did detect an elevation of autocrine EDIL3 by ELISA and western blot of CMs (Figure 4A). SMMC-7721 overexpressing EDIL3 exhibited a significant advantage of anoikis resistance and anchorage-independence compared with control cells (Figure 4B-E) without affecting the proliferation and invasion (Additional file 2: Figure S2C). Importantly, the addition of EDIL3 in control cells partly mimicked, albeit to a lower degree, these effect (Figure 4B-E). We obtained similar results in MHHC-LM3, a cell line that also exhibited a low basal level of autocrine EDIL3 (Additional file 4: Figure S4A-E).

We tested whether knocking down EDIL3 would cause the opposite effects. Using two shRNA sequences, we significantly suppressed the mRNA level of EDIL3, leading to a decrease in secreted EDIL3 in Huh-7 cells but no changes in intracellular EDIL3 in control cells (Additional file 5: Figure S5A). In agreement with the result from overexpression experiments, the decrease in EDIL3 in CM increased the caspase3/7 activity, lowered the WST-8 value and inhibited the colonies formed in soft agar, while adding recombinant EDIL3 partly rescued these inhibitory effects (Additional file 5: Figure S5B-D).

Taken together, these results indicate that EDIL3, especially secreted by cancer cells in our current study, is effective in protecting them from anoikis after suspension, thus supporting anchorage independent growth.

### EDIL3 promotes HCC tumorigenesis *in vivo*

**Figure 5:**
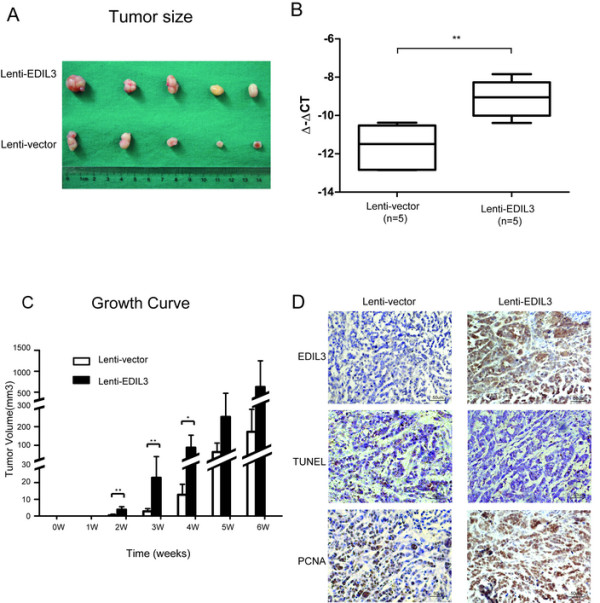
**EDIL3 promotes HCC tumorigenesis** ***in vivo.*** A total of 1.0 × 10^6^ of EDIL3-overexpressing or control SMMC-7721 cells are subcutaneously implanted into the right flank of 5 nude mice of each groups. The mice were observed and tumors formed are measured every week and resected after 6 weeks. **A**, After 6 weeks, EDIL3-overexpressing group shows relatively larger tumors compare with control group. **B**, qRT-PCR of 5 tumors in each group demonstrates a significant elevation in mRNA level. **C**, Tumor growth curve reveals a shorter latency in the EDIL3-overexpressing group (2 weeks) vs. the control group (4 weeks) and tumors in the EDIL3-overexpressing group are significantly larger than control group from 2^nd^ week to 4^th^ week. **D**, IHC stain in the same region of the tumor confirms the overexpression of EDIL3 and suggests more active proliferation (PCNA) and lower apoptosis (tunel) upon EDIL3 overexpression. **: P < 0.01.

To further elucidate the functional role of anoikis resistance conferred by EDIL3 *in vivo*, subcutaneous tumor formation assays with EDIL3-overexpressing and control SMMC-7721 were performed in nude mice. The results clearly indicate that the size of tumor formed in SMMC-7721 with EDIL3-overexpression is significantly larger than the control cells in the 2–4 week after subcutaneous implantation (Figure 5A, C). IHC staining and qRT-PCR confirmed the overexpression of EDIL3 in cancer cells (Figure 5B, C) and revealed that tumors with high EDIL3 exhibited a much lower apoptosis rate by TUNEL assay, whereas PCNA, a proliferative marker, was dominantly positive in EDIL3-overexpressing tumors but only sporadically positive in control tumors (Figure 5D). These results demonstrate that EDIL3 supports subcutaneous tumor formation.

### Activation of FAK-Src-AKT signaling by EDIL3 ligation is associated with anoikis resistance

**Figure 6:**
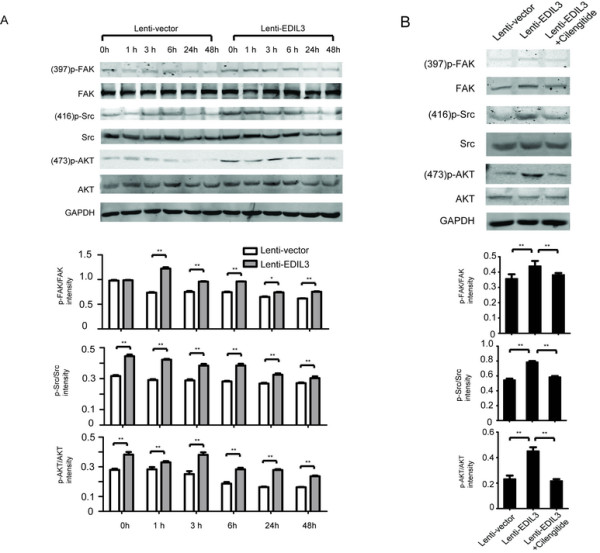
**Sustained activation of FAK-Src by EDIL3 through RGD recognition. A**, Western blot and densimetric analysis suggests EDIL3 overexpression sustains the signal intensity of FAK-Src and results in higher AKT phosphorylation within suspended SMMC-7721 cells over 48 hours. The elevated ^397^p-FAK, ^416^p-Src and ^473^p-AKT axis in overexpressing cells compare with control cells exists at most of the time points. **B**, Cilengitide (10 μM) reversed the activation of the FAK-Src-AKT axis induced by EDIL3. The signal intensity was examined after 24 h of suspension.

Because EDIL3 is a known ligand of integrins, we first examined whether HCC cell lines express receptors for EDIL3, such as integrin αV and α5. All of the cell lines used in this study expressed these integrins (Additional file 3: Figure S3B). We then examined the alteration of integrin-mediated signal pathways that were key to cell survival upon autocrine EDIL3. Indeed, we observed that EDIL3 activates signals downstream of integrins. During the 48 h of suspension, the pro-survival FAK-Src-AKT signal gradually faded away, suggesting the anoikis triggered by detachment. However, the FAK-Src-AKT signal was significantly sustained in EDIL3-overexpressing cells but tapered much more quickly in control cells (Figure 6A). The administration of recombinant EDIL3 also elevated these signal pathways, although to a lesser degree than overexpression (data not shown). However, pretreating cells with RDG-blocker cilengitide reversed the activation of FAK-Src-AKT (Figure 6B).

### Disrupting integrin ligation restores anoikis susceptibility to HCC

**Figure 7:**
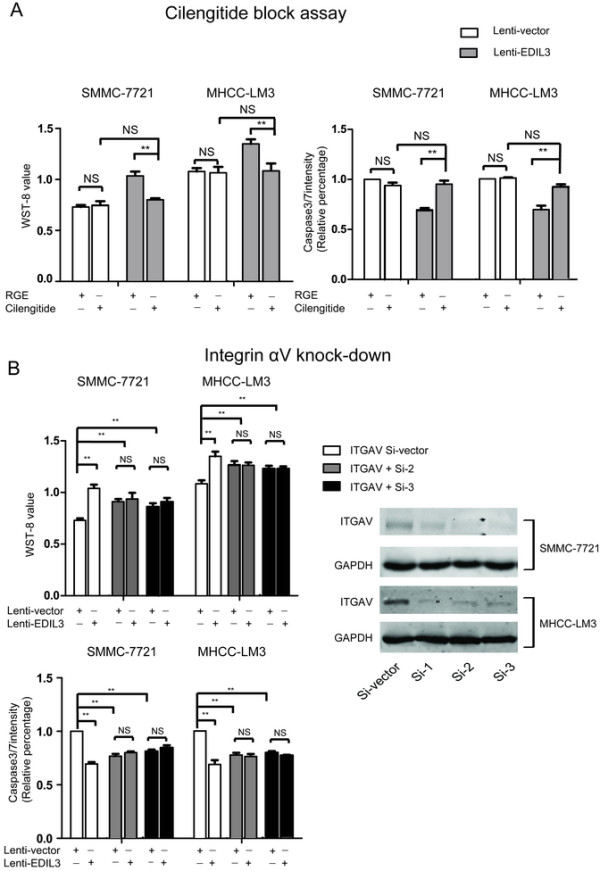
**Disrupting integrin-EDIL3 ligation deprives HCC of anoikis resistance induced by EDIL3 in SMMC-7721 and MHCC-LM3. A**, Cilengitide (10 μM) reduces the WST-8 value and increased casepase3/7 of the EDIL3 overexpressing group back to levels of control group, suggesting a blockage of EDIL3’s effect. There was not any effect observed in the control group in WST-8 and caspase3/7 assay. Both the two cell lines show consistent results. **B**, when integrin αV was knocked down to a very low level in both cell lines by two siRNAs (Si2 and Si3), the alteration in WST-8 and casepase3/7 value of EDIL3-overexpressing cells compared with control cells disappear. Interestingly, the WST-8 value and casepase3/7 intensity results suggest the overall anoikis significantly declined upon integrin αV silencing in both siRNA knock-down groups compare with si-control group. **: P < 0.01.

Based on the results above, we continued to examine weather disrupting the EDIL3/ integrins interaction could affect the anti-anoikis effect of EDIL3. In first effort, Cilengitide, an RGD-containing integrinαV antagonist, almost abrogated the anti-anoikis conferred by EDIL3 overexpression by lowering the WST-8 value (and increasing casepase3/7) to the control cells. However, Cilengitide did not result in an obvious pro-apoptotic effect on control cells, which have low EDIL3 expression (Figure 7A). In another effort, silencing of integrin αV by RNAi also led to a loss of protection of EDIL3. Surprisingly, the relative caspase3/7 intensity declined significantly with the down-regulation of integrin αV (Figure 7B); indeed, we observed similar results in an integrin α5 knock down assay (data not shown). These results suggest that RGD binding of integrinαV or integrinα5 is crucial for EDIL3-induced anti-anoikis and that integrins alone or when unligated might activate apoptosis in detached cancer cells.

## Discussion

This study provides a clear expression pattern of one ECM protein, EDIL3, in clinical samples and highlights the role of autocrine EDIL3 in the integrin-mediated interaction between HCC and ECM, which resulted in an anti-anoikis and anchorage-independent growth advantage. Our results also suggest a potential therapeutic strategy for personalized medicine by targeting ECM-integrin interactions for cancer cells in a subgroup of HCC patients.

In tissue level, EDIL3 is dominantly expressed and secreted by the endothelium, acting as a regulator of vascularization, immunocyte-endothelium adhesion and platelet microparticle clearance [14, 15, 26], or by a subset of macrophages to mediate engulfment [27]. Our data reveal that EDIL3 expression is turned on in adult human normal liver (cells) and is amplified during malignant transformation, which is different from mouse data, wherein EDIL3 is undetectable in the liver [15]. The cause of the up-regulation of EDIL3 in HCC is not clear; however, there is evidence suggesting that VEGF induces EDIL3 expression in malignant cells [28] and some inflammatory cytokines in endothelium [25]. Therefore it is possible that EDIL3 is elevated in response to cytokines in HCC. However, other events, such as HBV incorporation or epigenetic modifications, may also affect EDIL3 expression. Meaningfully, by analyzing an adequately sized sample, we demonstrated that when EDIL3 protein is accumulated to a high level, it is associated with larger tumor size and more PVTT formation, as well as greater relapse risk and shorter overall survival, which is consistent with a previous study, although the previous report only reached a positive result in overall survival [20], most likely due to the sample size and difference in patients selection.

In cell lines, however, EDIL3 demonstrates a unique expression. By examining multiple HCC cell lines and normal hepatocyte derived cell line, we find EDIL3 is expressed by both the normal and malignant cell lines, suggesting EDIL3 may not be directly linked to the relative tumorigenicity of HCC cell lines. Interestingly, we reveal that intracellular EDIL3 protein is observed at almost the same level despite the large differences in transcription. This inconsistency in transcription and translation was explained by the difference in secreted EDIL3. Moreover, either overexpressing or knocking-down the transcription level of EDIL3 only affected the secreted EDIL3. Based on these observations in cell lines and human samples, we postulate that EDIL3 is maintained in a standby status by many types of cells in quiescent (or normal) conditions and is secreted upon elevated transcription in physiologic or pathophysiological conditions; notably, the secretion is probably key to its biological functions. However, it will also be interesting to see the effects of EDIL3 knock-out in malignancies, and the potential regulation of EDIL3 secretion.

EDIL3 is well documented as an integrin ligand, modulating a wide range of biological processes that require integrins, such as integrin αV in angiogenesis and integrin αL in the neutrophil adhesion cascade [13, 29]. Because integrins such as integrin αV are also abnormally expressed in HCC [30], we postulated that EDIL3 may affect HCC cells through integrin ligation when it is secreted and anchored on the cell membrane, or deposited in the ECM. By overexpressing EDIL3 or recombinant EDIL3 treatment, we observed that secreted EDIL3 reduced the likelihood of anoikis in cancer cells and promoted anchorage-independent growth, both of which are indispensable for HCC spreading and PVTT formation, whereas do not affect proliferation or invasion. Indeed, all of the fresh PVTT samples in our study exhibited a very high level of EDIL3 protein, so it is highly possible that tumor cells bring this protective protein when leaving primary lesion, thus assisting PVTT formation. Similar strategies to overcome anoikis have been reported in other types of cancer, in which another ECM protein, collagen IV, is expressed [31].

The anoikis is a complex apoptosis process resulted from cell detachment from ECM or inappropriate cell-cell adhesion. The lack of ECM contact or the engagement with inappropriate ECM leads to the activation of anoikis from death receptors (extrinsic pathway) or mitochondria (intrinsic pathway) [32]. Integrins on the cell surface are crucial for anoikis modulation by modulating both the extrinsic and intrinsic pathway, passing opposite signals in their ligated or unligated status [21, 23, 33] or even independent of their ligation status [34]. The ^397^FAK-^416^Src-^473^AKT axis is a well-documented pro-survival pathway in anoikis resistance [32, 35–37]. Our results suggest that this axis is sustained when cancer cells are located in an environment rich with EDIL3, whereas phosphorylation of all three effectors was partly inhibited when RGD binding sites were blocked by cilengitide, suggesting that RGD recognition is critical for this pro-survival signal. Another interesting finding is that the suppression of integrin αV (or α5) expression led to a strong anoikis resistance, suggesting that unligated integrin may trigger anoikis, which is supported by other studies [38, 39]. However, intriguingly enough, integrin αV or α5 expression by cancer cells is required for invasion and spreading [40–42]. This seemly contradictory role of integrins reflects the importance of a suitable interaction between cancer cells and the microenvironment, or ECM, at the suitable stage of tumor progression; in this study, an EDIL3-integrin alliance may be exploited by detached HCC cells to escape anoikis when cells detach from the primary lesion, which resulted into a higher success rate of metastasis.

Finally, a variety or integrin inhibitors has entered clinical trial of different types of cancers [33, 43]. However, the outcomes have fallen short of expectations, revealing the complexity of integrin function as discussed above. Therefore, refined patient selection is required. Cilengitide was initially documented as an angiogenesis and invasion inhibitor [44]. However, our preliminary study suggests a potential role of cilengitide in inhibiting anoikis resistance in EDIL3-overexpressing HCC, thus pointing to a new potential indication of integrin inhibitors in HCC and providing a reference for patient selection.

## Conclusion

Our study suggests that high levels of autocrine EDIL3 may contribute to a receptive microenvironment for the survival of HCC cells and therefore assist cancer cell progression, which lead to adverse clinical results. Although other explanations may be concealed in our current effort, the anti-anoikis conferred by high level of EDIL3 is an important mechanism for through which EDIL3 provide protection for HCC cells. Disrupting the ligation of EDIL3 to integrins via RGD-blockers or inhibitors in selected patients with high EDIL3 may bear potential therapeutic value.

## Methods and materials

### Cell culture

The HCC cell line Sk-Hep1 was purchased from the American Type Cell Culture Collection (ATCC), HuH-7 was purchased from RIKEN Resource Centre, Japan, SMMC-7721 was purchased from the Cell Bank of the Chinese Academy of Sciences Cell Bank of Type Culture Collection. The metastatic HCC cell lines MHCC-97 L, MHCC-97H and MHCC-LM3 were obtained from the Liver Cancer Institute, Zhongshan Hospital, Fudan University. The HCC cell line CSQT-2 derived from a portal vein tumor thrombus (PVTT) has been described elsewhere [45]. Human liver epithelial cell line THLE-3 was purchased from ATCC;. All cell lines except THLE-3 were cultivated in DMEM (Dulbecco’s modified Eagle medium) supplemented with 10% (v/v) fetal calf serum at 37°C in a humidified incubator under 5% CO_2_ condition. THLE-3 was cultivated in BEGM (Lonza) with the addition of BEGM bullet kit according to the instruction on ACTT.

### Sample information

All samples were collected in Department of Liver Surgery, Renji Hospital, Shanghai Jiaotong University School of Medicine. Fresh samples, including tumor tissues and PVTTs, were obtained from HCC patients during tumor resection. Normal livers and cirrhotic livers were collected from healthy liver donors and cirrhosis patients, respectively, during liver transplantation. Approximately 202 HCC samples were collected from 2004 to 2010 and were constructed into tissue microarray (TMA). The median age of this cohort of patients was 50 years (range 17–73 years). The majority of patients are HBV-positive (187/202). The follow-up was ended in December 2012, and the median period was 33 months (range 2–90 months). All human samples were obtained with informed consent, and protocols were approved by the ethical review committee of the World Health Organization Collaborating Center for Research in Human Production (authorized by the Shanghai Municipal Government).

### Quantitative real-time PCR

Total RNA was extracted using Trizol reagent (Takara) and reverse transcribed using PrimeScript qRT-PCR kit (Takara) according to the protocol. Quantitative real-time PCR analyses were performed with SYBR Premix Ex Taq (Takara) on a 7300 Real-time PCR system (Applied Biosystems Inc.), and the primer for EDIL3 was as follows: forward, GTGAACTGTCGGGTTGTTCTGAG; and reverse, 5′-GGTTCCCAAGTGAACATGTCCAT-3′. The primers for ACTB were as follows: forward, 5′-TCACCCACACTGTGCCCATCTACGA-3′; and reverse, 5′-CAGCGGAACCGCTCATTGCCAATGG-3′. The relative expression of EDIL3 was analyzed by the comparative cycle threshold method (ΔΔCt method), which was normalized to ACTB.

### Protein collection, Western blot and antibodies

The HCC samples were handled with T-Per tissue protein extraction reagent (Thermo Scientific) according to the manufacturer’s protocol. Total cell protein was obtained by IP lysis buffer (Beyotime) for further assays. The secreted proteins in conditional media were collected by ethanol precipitation. Briefly, when cells grew to approximately 80% confluence, normal media were replaced by serum-free media. After 24 h, the media were collected, and 95% ethanol was added and kept overnight. The precipitated proteins were collected with SDS loading buffer and underwent standard western blot immediately. Western blot was performed using SDS-PAGE gel for proteins separation and nitrocellulose membrane for proteins blotting. The total volume of protein used in WB is 50 μg for tissue sample assays, and 30-60 μg in cell line assays. The signal was detected by the Odyssey infrared system (LI-COR).

The antibodies used were the following: EDIL3 (ProteinTech), ITGAV (Abcam), p-FAK^397^ (Cell Signal Technology), FAK (Abcam), p-Src^416^ (Epitomics), Src (Cell Signal Technology), p-AKT^473^ and AKT (Cell Signal Technology), StrepII (Abcam), GAPDH (ProteinTech) and β-actin (Abcam).

### Immunohistochemistry stain and analysis

A total of 202 samples of HCC on 2 TMA slices, 3 NLs, 10 HCCs and 6 PVTTs are subjected to IHC. Paraffin-embedded sections 5 μm thick and TMA were stained according to standard Immunohistochemistry (IHC) protocols. Heat-mediated antigen retrieval in pH 6.0 citric acid was performed. The antibodies used here were EDIL3 (ProteinTech). The scoring of EDIL3 was performed according to the ratio and intensity of positive-staining cells: 0-5% scored 0; 6-35% scored 1; 36-70% scored 2; and more than 70% scored 3. The final score was designated as negative (score 0), positive (score 1 or 2) and high positive (score 3). These scores were determined independently by two experienced pathologists in a blinded manner.

### Enzyme-linked immunosorbent assay

HCC cell lines were planted on 10 cm petri dish until 90% confluence, then were incubated in 3 ml serum free media for 48 hour. At the end of the incubation period, the conditional media were harvested and stored at -80C until use. The secreted EDIL3 in media were quantified using ELISA kits according to the manufacturer’s instructions (CUSABio).

### Immunofluorescence staining

In cell assay, all the HCC cell lines under tests were seeded on cover slides in 24-well plates and incubated overnight. For F-actin staining, cells were incubated with phalloidin-FITC (Sigma-Aldrich) for 75 minutes at room temperature. For EDIL3 staining, cells were incubated with primary antibodies against EDIL3 (Abnova) for 75 minutes at room temperature, followed by an Alexa Fluor 594-conjugated secondary antibody. In tissue staining, two samples embedded in paraffin were subjected to heat-mediated antigen retrieval in pH 6.0 citric acid, and blocked by 10% BSA. The primary antibodies used are EDIL3 (Abnova) and CD31 (Abcam) with Alexa Fluor 594-conjugated secondary antibody against EDIL3 and Fluor 488 against CD31. Immunofluorescence signals were captured using confocal micro-scopy (LSM 510, METALaser Scanning Microscope, Zeiss).

### Recombinant EDIL3 expression, purification and characterization

Human EDIL3 (NM_005711 Origene) with the signal peptide truncated were cloned into the episomal expression vector pCEP-Pu-Strep II-tag (N-terminal) in-frame with the sequence of the BM-40 (SPARC/osteonectin) signal peptide downstream of the CMV promoter, which has been described elsewhere [46]. Briefly, the expression vector was transfected into human embryonic kidney 293/Epstein-Barr nuclear antigen cells (EBNA-293) by Xetrem (Roche). The cells were screened with puromycin, and the surviving cells were cultured on a large scale. The culture medium was collected and centrifuged, and the supernatant was subjected to the StrepTactin sepharose column (IBA) for purification. The collected proteins were quantified and validated by Coomassie Brilliant Blue (CBB) staining and western blot. The empty vector was applied as control proteins.

### Establishment of stable overexpression or knock-down cell lines

For the overexpression of EDIL3 in cell lines, the full-length cDNA of EDIL3 (NM_005711 Origene) was subcloned into pCDH-CMV-MCS-EF1-Puro vector (System Biosciences). Lenti-virus was packaged in 293 T cells using Lipofectamine2000 (Invitrogen), and the virus DNA was transduced into cell lines. For knock-down of EDIL3, short hairpin RNA (shRNA) sequences (Sh1:5′-CCGGCCCAAGTTTGTCGAAGACATTCTCGAGAATGTCTTCGACAAACTTGGGTTTTTG- 3′; Sh2: 5′-CCGGGGAGGTTGCATCAGATGAAGACTCGAGTCTTCATCTGATGCAACCTCCTTTTTG- 3′) targeting EDIL3 were cloned into pLKO.1-puro vectors (Roche). ShRNA-containing plasmids were packaged into lenti-viruses and transduced into target cell lines as above. The efficiency of over-expression or knock-down was tested by qRT-PCR and western blot.

### Anoikis assay

Anoikis was induced by culturing cells in poly-HEMA coated plates as described by others [47]. Briefly, poly-HEMA were prepared as a 10 mg/ml solution in ethanol, which covered completely the petri-dish or plates, then dried and repeated once. Cells in serum-free medium were seeded into the coated plates with or without adding the recombinant EDIL3 at the corresponding concentration. To avoid survival effects caused by the clumping of cells, 0.5% methyl cellulose (Sigma-Aldrich) was added into the medium. At the designated time points, the suspended cells were collected and subjected to cell viability assays by WST-8 kit (Dojindo), apoptosis assays by Caspase3/7 Glo kit (Promega), and Annexin V/PI staining on FACS (BD) according to the protocols from the respective manufacturers.

### Anchorage-independent growth assay

Colony formation in soft agar was tested to assay anchorage-independent growth. Stable overexpressing EDIL3 cell lines SMMC-7721 and MHCC-LM3 or their control cells were suspended in the upper layer consisting of culture medium with 1% FBS and 0.35% agar, which is above a basal layer of 0.6% agarose in 6-well plates in a triplicate manner. The cell density was 2,500 cells per well. The soft agars were fed twice a week with 0.3 ml of culture medium. In recombinant EDIL3 treatment assay, the soft agars were fed every 2 days with serum-free culture medium containing EDIL3 or empty vector. Colonies were stained with 0.05% crystal violet, and all the visible colonies were counted by microscopy after 21–28 days.

### Tumorigenesis in vivo

A total of 1.0 × 10^6^ of lenti-vector or lenti-EDIL3 SMMC-7721 cells were implanted subcutaneously into the right flank of 5 BALB/c (nu/nu) mice in each group. Tumor sizes were measured once a week, and mice were sacrificed for the analysis of tumor burden after 6 weeks. All procedures were performed in accordance with The Animal Care and Use Committee of Jiaotong University. The resected mice tumors were fixed in methanol and embedded in paraffin before being subjected to IHC using anti-EDIL3 (ProteinTech), anti-PCNA (Abcam) and anti-TUNEL (Millipore).

### RGD-blocking assay and knock-down of ITGAV

Cyclic RGD-containing peptide cilengitide (Selleckchem) or control peptide RGE was used to block the ligation of EDIL3 to integrins on the surface of HCC cells at a final concentration of 10 μM. Briefly, the cells was suspended in Poly-HEMA coated plates and exposed to the peptides about 3 hours after suspension. Then, the cells were subjected to the assays as described above. SiRNA targeting ITGAV (Si2: 5′-GCCAGCCAAUUGAAUUUGATT- 3′; Si3: 5′-CCCAGUUGUAUCUCACAAATT - 3) was used to knock down the protein level of this integrin and was confirmed by western blot. After 72 h of transfection, the cells were subjected to the assays as above.

### Statistical analysis

Statistical analyses were performed with SPSS 16.0 software. The association between EDIL3 expression and clinicopathological parameters was analyzed by Pearson Chi-Square test. Overall survival and relapse risk was calculated by the Kaplan-Meier method and compared by the log-rank test. Statistical significance was determined by two-tailed Student’s *t* test for any differences observed (P < 0.05).

## Electronic supplementary material

Additional file 1: Figure S1: A, The protein level of EDIL3 in NL, CL, HCC and PVTT run on the same gel was quantified by EDIL3/β-actin densitometry analysis respectively. The density of NL or CL samples are significantly lower compare with HCC and PVTT. B, Immunofluorescence staining of EDIL3, F-actin and DAPI in confocal microscope shows EDIL3 is localized within cells and at almost the same intensity in the cell lines. (TIFF 2 MB)

Additional file 2: Figure S2: The EDIL3 is widely expressed in other cancer cell lines. A, The EDIL3 expression pattern in 6 pancreatic cancer, 5 gastric and 2 endometrial carcinoma cell lines showed a difference mRNA level, while the protein level within cell was almost the same Neither EDIL3 overexpression or administration affects the invasion or proliferation of HCC cells. B,C, Neither administration of recombinant EIDL3 or EDIL3 overexpression led to a change of proliferation or invasion of SMMC-7721, which are examined by WST-8 assay and transwell-matrigel invasion assay respectively. Lenti-EDIL3-E2 stands for monoclonal overexpressing cells. (TIFF 2 MB)

Additional file 3: Figure S3: A. Validation of purified recombinant EDIL3-StrepII protein by coomassie blue and western blot. Coomassie blue show a single brand, which validate the purity of protein. Both the EDIL3 antibody and StrepII antibody detect a single brand, and the intensity is consistent with concentration, which further validate the accuracy of the result. B, All the normal or HCC cell lines express integrinαV as shown in western blot. (TIFF 905 KB)

Additional file 4: Figure S4: EDIL3 overexpressed by MHCC-LM3 significantly increases the anoikis resistance and anchorage independence. A, overexpression of EDIL3 is validated by qRT-PCR, western blot and ELISA, confirming the increase in transcription leads to a higher autocrine EDIL3 whereas no change within cell. B, EDIL3 overexpressing MHCC-LM3 formed more clones in soft agar compared with control cells, which can be partly mimicked by recombinant EDIL3 (50nM). C, After being suspended for 72 h, more cells in overexpressing group survived compared with control cells. D, caspase3/7 intensity assay validated the difference in survival between two groups was caused by lower apoptosis. E, Annexin V/PI stain by FACS was performed to validate the apoptosis result. *: P < 0.05; **: P < 0.01. (TIFF 2 MB)

Additional file 5: Figure S5: Knocking down EDIL3 in Huh-7 increases the anoikis and suppresses the anchorage-independent growth. A, knocking down of EDIL3 by 2 shRNA was validated by real-time PCR, western blot and ELISA, confirming the suppression in transcription leads to a lower autocrine EDIL3. B, after being suspended for 48 h, both two knock-down group showed a less survived cell compared with control group. C, caspase3/7 intensity assay validated the difference in survival is due to more apoptosis in knock-down group. E, Annexin V/PI stain by FACS was performed to validate the apoptosis result. *: P < 0.05; **: P < 0.01. (TIFF 2 MB)
