# Supplementary figures and images for: Elevated autocrine EDIL3 protects hepatocellular carcinoma from anoikis through RGD-mediated integrin activation
Source: Mol Cancer. 2014 Oct 1;13:226. doi: 10.1186/1476-4598-13-226 (PMC4200221; doi:10.1186/1476-4598-13-226)

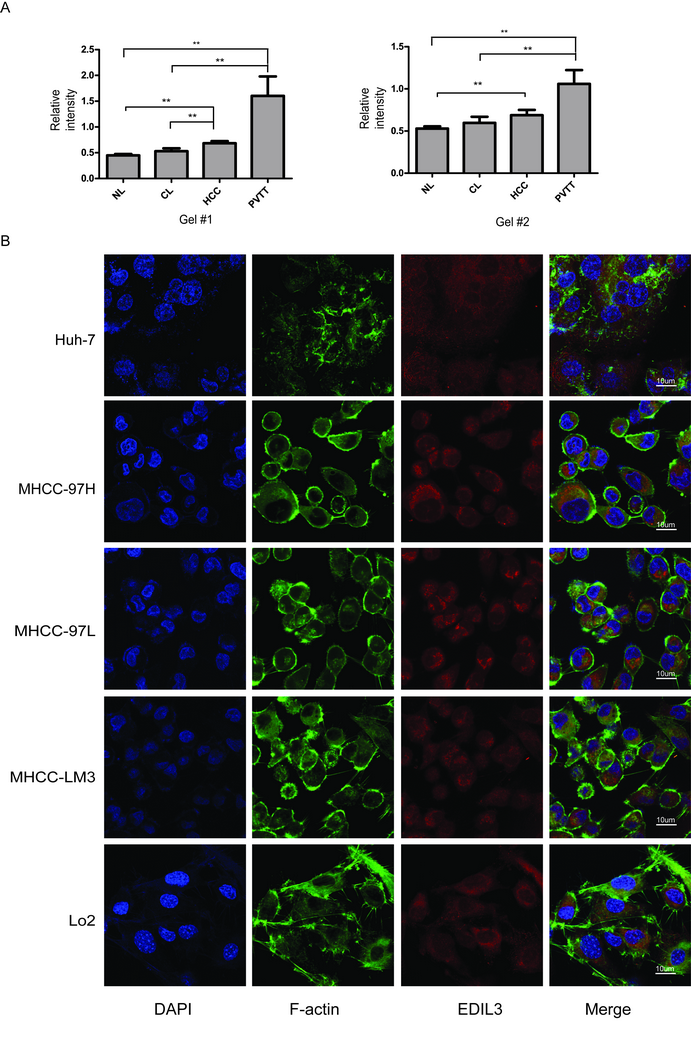

Supplement: Supplementary file 1 — Additional file 1: Figure S1: A, The protein level of EDIL3 in NL, CL, HCC and PVTT run on the same gel was quantified by EDIL3/β-actin densitometry analysis respectively. The density of NL or CL samples are significantly lower compare with HCC and PVTT. B, Immunofluorescence staining of EDIL3, F-actin and DAPI in confocal microscope shows EDIL3 is localized within cells and at almost the same intensity in the cell lines. (TIFF 2 MB) [file 12943_2014_1431_MOESM1_ESM.tiff]

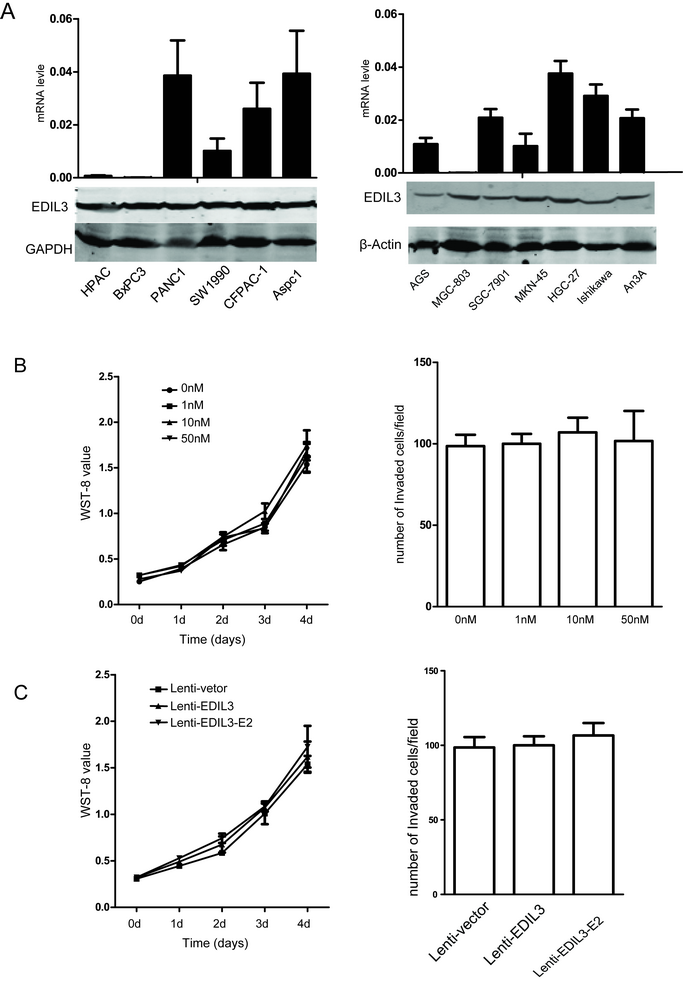

Supplement: Supplementary file 2 — Additional file 2: Figure S2: The EDIL3 is widely expressed in other cancer cell lines. A, The EDIL3 expression pattern in 6 pancreatic cancer, 5 gastric and 2 endometrial carcinoma cell lines showed a difference mRNA level, while the protein level within cell was almost the same Neither EDIL3 overexpression or administration affects the invasion or proliferation of HCC cells. B,C, Neither administration of recombinant EIDL3 or EDIL3 overexpression led to a change of proliferation or invasion of SMMC-7721, which are examined by WST-8 assay and transwell-matrigel invasion assay respectively. Lenti-EDIL3-E2 stands for monoclonal overexpressing cells. (TIFF 2 MB) [file 12943_2014_1431_MOESM2_ESM.tiff]

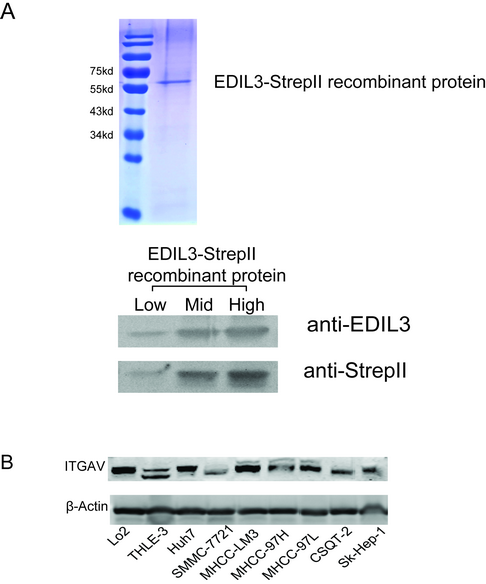

Supplement: Supplementary file 3 — Additional file 3: Figure S3: A. Validation of purified recombinant EDIL3-StrepII protein by coomassie blue and western blot. Coomassie blue show a single brand, which validate the purity of protein. Both the EDIL3 antibody and StrepII antibody detect a single brand, and the intensity is consistent with concentration, which further validate the accuracy of the result. B, All the normal or HCC cell lines express integrinαV as shown in western blot. (TIFF 905 KB) [file 12943_2014_1431_MOESM3_ESM.tiff]

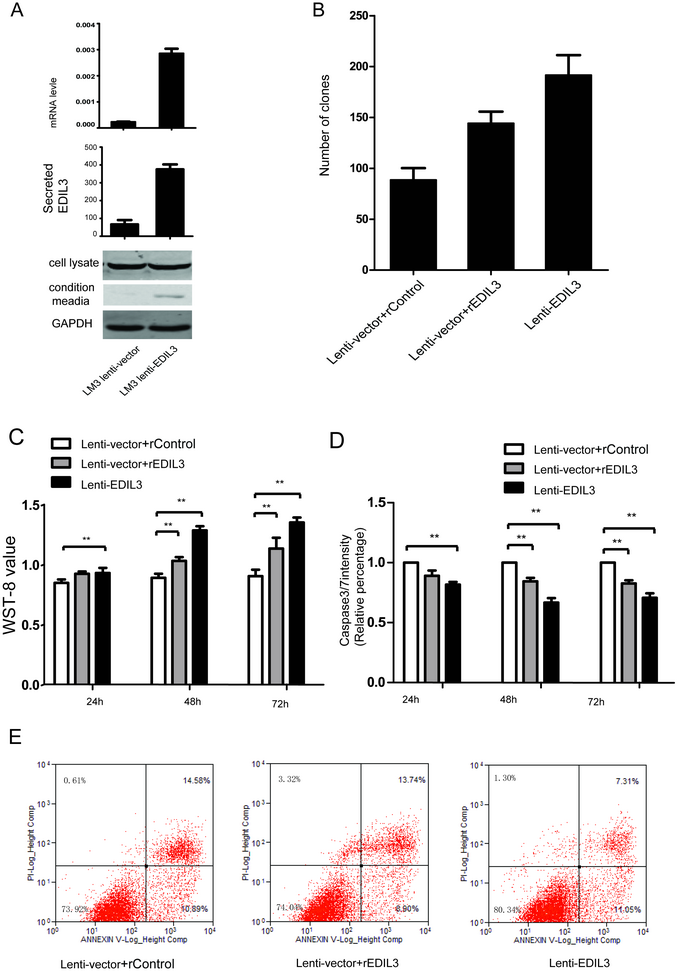

Supplement: Supplementary file 4 — Additional file 4: Figure S4: EDIL3 overexpressed by MHCC-LM3 significantly increases the anoikis resistance and anchorage independence. A, overexpression of EDIL3 is validated by qRT-PCR, western blot and ELISA, confirming the increase in transcription leads to a higher autocrine EDIL3 whereas no change within cell. B, EDIL3 overexpressing MHCC-LM3 formed more clones in soft agar compared with control cells, which can be partly mimicked by recombinant EDIL3 (50nM). C, After being suspended for 72 h, more cells in overexpressing group survived compared with control cells. D, caspase3/7 intensity assay validated the difference in survival between two groups was caused by lower apoptosis. E, Annexin V/PI stain by FACS was performed to validate the apoptosis result. *: P < 0.05; **: P < 0.01. (TIFF 2 MB) [file 12943_2014_1431_MOESM4_ESM.tiff]

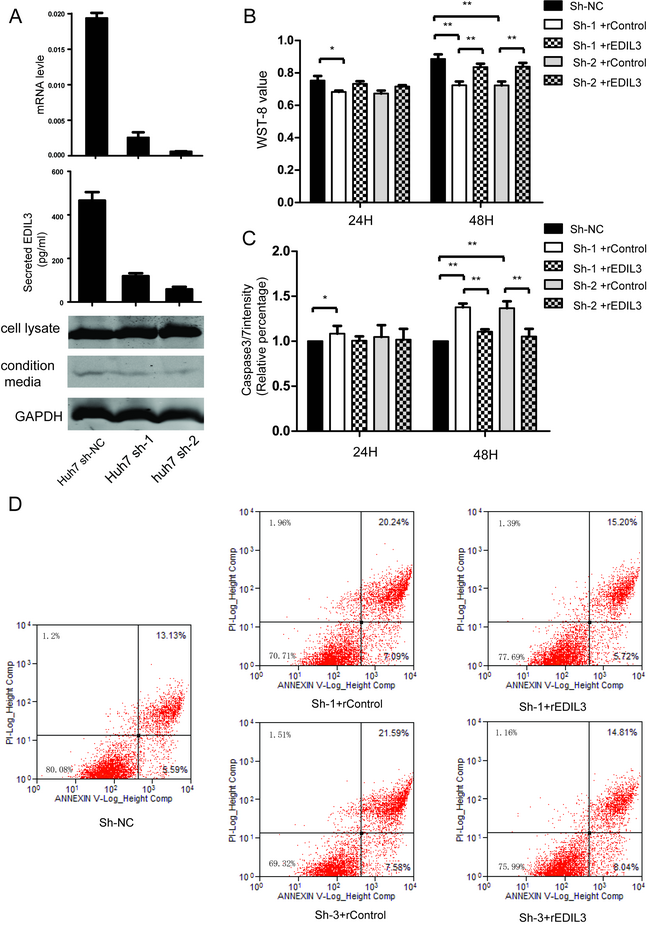

Supplement: Supplementary file 5 — Additional file 5: Figure S5: Knocking down EDIL3 in Huh-7 increases the anoikis and suppresses the anchorage-independent growth. A, knocking down of EDIL3 by 2 shRNA was validated by real-time PCR, western blot and ELISA, confirming the suppression in transcription leads to a lower autocrine EDIL3. B, after being suspended for 48 h, both two knock-down group showed a less survived cell compared with control group. C, caspase3/7 intensity assay validated the difference in survival is due to more apoptosis in knock-down group. E, Annexin V/PI stain by FACS was performed to validate the apoptosis result. *: P < 0.05; **: P < 0.01. (TIFF 2 MB) [file 12943_2014_1431_MOESM5_ESM.tiff]
